# Low dietary oyster mushroom spent substrate limitedly ameliorates detrimental effects of feeding combined marula seed cake and mucuna seed meal as soya bean replacements in broiler chickens

**DOI:** 10.1007/s11250-023-03878-9

**Published:** 2024-01-09

**Authors:** Makiwa Simeon Mthana, Doctor Mziwenkosi Nhlanhla Mthiyane

**Affiliations:** 1 Department of Animal Science, School of Agricultural Sciences, Faculty of Natural and Agricultural Sciences, North-West University (Mahikeng Campus), Private Bag X 2046, Mmabatho, 2735 South Africa; 2 Food Security and Safety Focus Area, Faculty of Natural and Agricultural Sciences, North-West University (Mahikeng Campus), Mmabatho, 2735 South Africa

**Keywords:** Broiler, Growth performance, Marula seed cake, Mucuna seed meal, Mushroom substrate

## Abstract

This study investigated ameliorative effects of dietary oyster mushroom (*Pleurotus ostreatus*) spent substrate (OMSS) in broiler chickens fed diets supplemented with combined marula seed cake (MSC) and mucuna seed meal (MSM) replacing soya bean meal (SBM). In a completely randomised design (CRD), 400 day-old Ross 308 chicks were randomly allocated to 5 iso-nitrogenous-energetic diets (control with 100% SBM, control with 60% MSC and 40% MSM replacing SBM (MSC + MSM), MSC + MSM with 1.25% OMSS, MSC + MSM with 2.5% OMSS, and MSC + MSM with 5% OMSS) each with 8 replicate pens of 10 during starter, grower and finisher phases. Dietary MSC + MSM decreased (*P* < 0.001) feed intake (FI), body weight gain (BWG), and feed conversion efficiency (FCE); slaughter weight, hot carcass weight (HCW), cold carcass weight (CCW), breast weight, and back lengths (*P* < 0.001); serum SDMA and alanine transaminase (*P* < 0.05). In contrast, it increased the weights of the thigh (*P* < 0.001), wing (*P* < 0.01), liver (*P* < 0.001), proventriculus (*P* < 0.001), gizzard (*P* < 0.001), duodenum (*P* < 0.001), jejunum (*P* < 0.001), ileum (*P* < 0.001), and caecum (*P* < 0.01) and serum alkaline phosphatase (*P* < 0.05) and cholesterol (*P* < 0.01). Further, it increased meat redness and decreased its hue angle at 45 min post-slaughter (*P* < 0.01) whilst it decreased its pH (*P* < 0.01) and increased its shear force (*P* < 0.05) at 24 h post-slaughter. Compared to higher levels, low (1.25%) dietary OMSS improved, though limitedly, FI, BWG, and FCE at grower and finisher phases only (*P* < 0.001) whilst it reversed MSC plus MSM-induced deleterious effects on slaughter weight, HCW, and CCW (*P* < 0.001) and increases in gizzard weight (*P* < 0.001) and meat shear force at 24 h post-slaughter (*P* < 0.05). Otherwise, OMSS generally decreased (*P* < 0.05) serum SDMA and alanine transaminase whilst it abrogated and augmented increases in serum alkaline phosphatase (*P* < 0.05) and cholesterol (*P* < 0.01), respectively, and reversed the increase and decrease in meat redness (*P* < 0.01) and hue angle (*P* < 0.05), respectively. In conclusion, dietary replacement of SBM with combined MSC plus MSM induced deleterious effects in broiler chickens that were limitedly abrogated by low (1.25%) inclusion level of OMSS.

## Introduction

Formidable improvements in the genetics and nutrition of broiler chickens that led to their massive meat yields over short production cycles have positioned these birds as the most strategic animal production sector to sustainably achieve security of protein-rich food to nourish the rapidly growing global human population in a relatively environmentally friendlier way (Liebl et al. 2022). Indeed, broiler chickens are at the top of annual meat production forecasts worldwide (Research and Market 2019), with their meat being the most consumed animal-derived meat globally (Henchion et al. 2017; FAO 2020). To achieve their massive level of productivity, these modern birds are conventionally fed nutrient-dense diets mainly rich in energy and protein, with SBM predominantly used globally to supply the latter nutrient consequent to its high crude protein (CP) content and balanced amino acid profile (Hejdysz et al. 2019). Unfortunately, the price of SBM frequently fluctuates worldwide with a tendency to burgeon with dwindling supply at certain periods of the year (Shi et al. 2012). Also, aside soya bean utilisation for biofuel production (Pimentel et al. 2008; Attia et al. 2014), its use as human food worsens the stiffness of the human food-animal feed competition (Al-Sagan et al. 2020). In addition, the pulse is mainly produced in the Americas with Brazil (33%), the USA (28%), and Argentina (16%) responsible for approximately 80% of its total global production (De Maria et al. 2020) and most countries particularly in the developing world incurring enormous costs of its importation. These factors result in exorbitant costs of broiler chicken feeds reported to represent approximately 65–75% of total variable costs of producing the birds (Panda et al. 2014; Mallick et al. 2020). Moreover, intensification and expansion of agricultural lands for soya bean production not only degrade the environment but also incur costs of deforestation and natural vegetation clearance (Sauer 2018). Together, these factors accentuate the need for exploration of cost-effective and environmentally sustainable alternative protein sources (APS) such as MSC and MSM for replacement of SBM in broiler diets.

An industrial by-product of oil extraction from marula (*Sclerocarya birrea* subsp. *caffra*) seed kernels, MSC, is similar to SBM in its CP content (470 g/kg DM) (Mlambo et al. 2011; Mdziniso et al. 2016) and profile of essential and non-essential amino acids with substantial residual oil that is rich in *n*-9 monounsaturated fatty acid (MUFA) oleic acid (72% to 85%) (Mthiyane and Mhlanga 2017). Among non-ruminant animals, it has been investigated as an alternative to SBM in the diets of Japanese quails (Mazizi et al. 2020), pigs (Malebana et al. 2018; Thabethe et al. 2022), and broiler chickens (Manyeula et al. 2022; Mthana et al. 2023). Nutritionally, the major limitation of MSC is its deficiency of the essential amino acid lysine (Mthiyane and Mhlanga 2017; Malebana et al. 2018), the second most limiting amino acid in maize-SBM-based diets after the sulphur amino acid methionine (Dozier et al. 2009). Dietary lysine is necessary for improved growth performance and meat quality in broiler chickens (Da Costa et al. 2017; Tian et al. 2017; Sharma et al. 2018), with its deficiency constraining body weight and feed intake (Bastianelli et al. 2007), among other things.

On the other hand, in addition to its abundance in CP (25–35 g/100 g) and starch (39–41 g/100 g) (Ezegbe et al. 2023), the tropical/sub-tropical legume mucuna [*Mucuna pruriens* (L.) DC var. *utilis* (Wall. ex Wight) Baker ex Burck (a.k.a. velvet bean)] seed meal (MSM) is rich in lysine and other essential amino acids including methionine (Kouakou et al. 2022). Hence, its combination with MSC in replacement of SBM would be envisaged to balance the profile of lysine and other essential amino acids, in addition to supplying energy, to broiler chicken diets. Indeed, many studies have embraced this concept of blending various protein sources as alternatives to SBM as a strategy to take advantage of their complementarity in supplying amino acids, other nutrients and beneficial bioactive compounds to diets of broiler chickens and other poultry species (Min et al. 2009; Leiber et al. 2017; Liebl et al. 2022).

Notwithstanding, both MSC and MSM are fraught with potentially toxic chemical substances that serve as antinutritional factors (ANFs) in broiler nutrition. In this connection, aside its low levels of condensed tannins and other phytochemicals (Malebana et al. 2018; Mthana et al. 2023), MSC’s high content of CP and energy predisposes it to infestation with mycotoxins including deoxynivalenol (DON) and T-2 toxin and its residual oil can harbour lipid peroxides (Mthiyane and Mhlanga 2017), depending on storage conditions. Similarly, MSM is naturally endowed with numerous ANFs primary among which is a high content of the toxic amino acid 3,4-dihydroxy-L-phenylalanine (L-DOPA) (Bhat 2011; Cacabelos 2017) as well as a high fibre content (9.7–19.3%) (Belewu and Olajide 2010). Whilst some of these ANFs can impart beneficial effects in animals especially at low dietary levels including antimicrobial and antioxidant attributes of condensed tannins (Bharadwaj et al. 2021) as well as L-DOPA’s muscle building (Navegantes et al. 2000), hypoglycemic, hypolipidemic, anti-cholesterolemic (Dharmarajan and Arumugam 2012; Ngatchic et al. 2016), and meat quality enhancing (Yantika et al. 2016) properties, these chemical substances can induce detrimental effects in broiler chickens. Indeed, previous studies have shown that dietary intake of mycotoxins DON and T-2 toxin (Huff et al. 1986; Kubena et al. 1988, 1989), lipid peroxidised oils (Engberg et al. 1996), and L-DOPA (Del Carmen et al. 1999; Vadivel and Pugalenthi 2010) can induce performance and meat quality perturbations in the modern birds. Also, a high dietary content of fibre decreases the digestibility of feed (Ding et al. 2012). Consequently, there is a need for a strategy to abrogate untoward effects of ANFs in broiler chicken diets supplemented with MSC and MSM.

The detrimental effects of most ANFs including mycotoxins (Adhikari et al. 2017) and L-DOPA (Rahal et al. 2014) are mediated through oxidative stress in birds and other animals. This has been manifested through mycotoxin-induced decrease in antioxidant enzyme activities in various tissues (Hou et al. 2013). Also, L-DOPA-induced oxidative stress has been demonstrated through production of toxic free radicals (Corona-Morales et al. 2000), protein carbonyls, and advanced glycation end-products (Nikolova et al. 2019) resulting in mitochondrial dysfunction, DNA damage (Dias et al. 2013), liver inflammation and general cytotoxicity (Ala-Kurikka et al. 2019). These aberrant manifestations suggest that any strategy to ameliorate mycotoxin and L-DOPA-induced toxicity in broiler chickens fed MSC and MSM supplemented diets should involve dietary supply of antioxidants to quench oxidative stress and biodegradative enzymes to break down toxic biomolecules that have capacity to trigger the stress. In this regard, the spent substrate of oyster mushrooms (*P. ostreatus*; OMSS) is a rich source of antioxidant bioactive compounds and biodegradative enzymes with enormous potential to biodegrade toxic ANFs including mycotoxins (Jackson and Pryor 2017; Branà et al. 2020) as well as fibre particularly lignin (Santhanam et al. 2011; Mate and Alcalde 2017). In this connection, the mycelium of oyster mushrooms produces a plethora of enzymes such as lignin peroxidase, manganese peroxidase, and laccase (Nyanhongo et al. 2007) that have the capacity to biodegrade toxic and fibrous substances in biological materials (Roach et al. 2013). Against this background, it was hypothesised that incorporation of OMSS into broiler chicken diets supplemented with MSC and MSM replacing SBM would improve the safety of the feed, thus enhancing the growth performance, health, meat quality and liver-gut-serophysio-biochemistry of broiler chickens.

## Method and materials

### Study site

The study was conducted at North-West University Molelwane Experimental Farm located (coordinates: 25°40.459′S, 26°10.563′E) approximately 8 km outside Mahikeng City towards Ramatlabama border post to Botswana, in the Mahikeng Local Municipality of Ngaka Modiri Molema District, North-West Province, South Africa.

### Sourcing and preparation of research materials

Oyster mushroom spawn was supplied by Eco-Agro Enterprise (Pty) Ltd. (Mbombela, Mpumalanga Province, South Africa) whereas MSC was obtained from The Marula Company in Phalaborwa (Limpopo Province, South Africa) and the black-coated *M. pruriens utilis* seeds from AGT Foods (Pty) Ltd. (Krugersdorp, Gauteng Province, South Africa). The mucuna seeds were then milled (screen size 1 mm) and kept in 50 kg bags in the feed shed. All other dietary ingredients were supplied by SimpleGrow (Pty) Ltd. (Centurion, Gauteng Province, South Africa).

### Oyster mushroom spent substrate production

Sunflower husks, the substrate used for oyster mushroom cultivation, were soaked in water for 4 h, sterilised for 1 h in a laboratory autoclave, and then cooled at room temperature. The sterilised substrate was then transferred into sterile polyethylene mushroom bags and inoculated with 300 g/kg oyster mushroom spawn in 5 replicates. The replicate bags with inoculated substrate were perforated to allow gas exchange and kept in a dark incubation room with average temperature maintained at 29.4 ℃ and relative humidity between 75 and 80% for 35 days to allow mycelial colonisation of the substrate. In the morning of day 36, the bags were opened and the OMSS dried under shade. Upon drying, the OMSS was milled (1 mm) and stored in polyethylene bags at room temperature pending chemical analysis.

### Chemical analysis

Samples of MSC, MSM, and OMSS were milled (1 mm) and analysed (Table 1) for dry matter (DM) (method 930.15), CP (method 954.01), ether extract (EE) (method 920.39), and ash (organic matter, OM) (method 942.05) following AOAC (2005) procedures as well as for neutral detergent fibre (NDF), acid detergent fibre (ADF), and acid detergent lignin (ADL) (Van Soest et al. 1991). The DM was determined by putting 1 g of sample into pre-weighed crucibles and oven-drying it at 105 ℃ for 12 h after which it was placed in a desiccator for cooling and then weighed. The CP content was determined following the Kjeldahl method by analysing the nitrogen content of each sample and multiplying it by a factor of 6.25. The EE was determined by extracting crude fat with petroleum ether using an automated Soxhlet Fat Analyzer ANKOM^XT15^ extractor following the operator’s manual (ANKOM Technology, Macedon, NY, USA). The ash content was determined by igniting a dry sample (1 g) in the muffle furnace (Nabertherm GmbH, Germany) for 6 h at 550 ℃. Then, the OM content was calculated by subtracting the % ash from 100%. The NDF and ADF contents were analysed using an automated ANKOM^DELTA^ fibre analyser (ANKOM Technology, Macedon, NY, USA). The ADF residue bags were then immersed in 72% (wt/wt) H_2_SO_4_ for 3 h to determine the ADL content. The hemicellulose content was then calculated by subtracting the % ADF from % NDF. Non-fibre carbohydrates (NFC) were calculated by subtracting NDF, CP, and EE from OM as described by De Bellis et al. (2022). Digestible energy (MJ/kg) was estimated using the following standard formula: DE = 17.47 + (0.0076 × CP g/kg) + (0.0158 × EE g/kg) − (0.0331 × ash g/kg) − (0.0140 × NDF g/kg) adopted from McDonald et al. (2011). For mineral analysis, the samples were digested using an automated sample digester [Titan Microwave Sample Preparation System, PerkinElmer (Pty) Ltd.]. Both the macro and micro minerals were then analysed using an ICP Mass Spectrometer (NexION® 2000, PerkinElmer (Pty) Ltd.).

**Table 1:** Chemical composition of MSC, MSM, and OMSS (g/kg DM, unless stated otherwise)

Parameter	MSC	MSM	OMSS
Dry matter	938.70	912.70	870.00
Crude protein	471.80	232.69	66.50
Ether extract	168.24	84.52	13.92
Ash	75.12	34.24	35.70
Organic matter	924.88	965.76	964.30
Neutral detergent fibre	122.14	368.60	424.61
Acid detergent fibre	62.33	246.29	350.12
Acid detergent lignin	27.45	71.81	241.11
Hemicellulose	59.80	122.40	74.44
Non-fibre carbohydrates	162.80	279.98	459.27
Digestible energy (MJ/kg)	19.52	14.27	11.13

*MSC* marula seed cake, *MSM* mucuna seed meal, *ND* not detected, *OMSS* oyster mushroom spent substrate

### Diet formulation

Five maize-SBM-based iso-nitrogenous and iso-caloric broiler diets were formulated, with and without MSC plus MSM completely replacing SBM and graded levels (1.25%, 2.5% and 5%) of OMSS, to meet the nutrient requirements of broiler chickens at starter (d1–14), grower (d15–28), and finisher (d29–42) phases (NRC 1994) (Table 2). The inclusion levels of MSC (~ 15%) (Mthana et al. 2023) and MSM (~ 10%) (Mthana and Mthiyane 2023; Zungu et al. 2023) were based on optimum inclusion levels for these APS observed in our preliminary studies. Hence, these APS were included in the diets at 60% and 40% (MSC plus MSM) to completely replace SBM (i.e. Diet 1 contained 100% SBM, Diet 2 with 60% MSC plus 40% MSM, Diet 3 with 60% MSC plus 40% MSM + 1.25% OMSS, Diet 4 with 60% MSC plus 40% MSM + 2.5% OMSS, and Diet 5 with 60% MSC plus 40% MSM + 5% OMSS).

**Table 2:** Ingredients and nutrient composition (g/kg on as-fed basis, unless stated otherwise) of starter (d1–14), grower (d15–28) and finisher (d29–42) diets

	Starter					Grower					Finisher				
			MSC + MSM					MSC + MSM					MSC+MSM		
	SBM	Only	+1.25SMS	+2.5SMS	+5SMS	SBM	Only	+1.25SMS	+2.5SMS	+5SMS	SBM	Only	+1.25SMS	+2.5SMS	+5SMS
Ingredient (g/kg)															
Yellow maize	576.7	313.6	318.8	319.3	328.9	628.2	457.1	480.6	467.8	476.4	648.0	539.9	545.2	550.3	557.2
Pellibond	10.0	10.0	10.0	10.0	10.0	10.0	10.0	10.0	10.0	10.0	10.0	10.0	10.0	10.0	10.0
Full fat soya	0.0	0.0	0.0	0.0	0.0	43.0	0.0	0.0	0.0	0.0	101.2	0.0	0.0	0.0	0.0
Soya bean meal (46.5% CP)	300.2	0.0	0.00	0.0	0.0	225.5	0.0	0.0	0.0	0.0	165.9	0.0	0.0	0.0	0.0
MSC	0.0	157.5	157.5	160.0	160.0	0.0	150.0	141.2	150.0	151.3	0.0	140.0	140.0	140.0	142.5
MSM	0.0	100.0	100.0	100.0	100.0	0.0	90.0	90.0	90.0	90.0	0.0	80.0	80.0	80.0	80.0
Sunflower oilcake (32% CP)	57.4	138.4	141.8	135.7	140.0	28.5	67.0	109.8	74.8	78.7	23.3	83.1	86.7	90.1	90.5
Wheat bran	0.0	238.1	216.9	210.0	174.4	0.0	190.2	105.9	145.7	106.6	0.0	123.9	102.6	81.4	47.0
OMSS	0.0	0.0	12.5	25.0	50.0	0.0	0.0	12.5	25.0	50.0	0.0	0.0	12.5	25.0	50.0
Crude soya oil	15.7	0.0	0.0	0.0	0.0	30.0	0.0	0.0	0.0	0.0	30.0	0.0	0.0	0.0	0.0
L-Lysine HCl	3.6	6.4	6.4	6.4	6.4	3.6	5.7	6.0	5.9	6.0	3.2	5.3	5.3	5.4	5.5
DL-Methionine	3.0	3.3	3.4	3.4	3.5	3.2	3.5	3.5	3.5	3.6	2.2	2.4	2.4	2.5	2.6
L-Threonine	1.2	2.3	2.2	2.4	2.4	1.2	2.0	2.0	2.1	2.2	0.7	1.4	1.4	1.5	1.5
L-Tryptophan	0.0	0.01	0.03	0.04	0.1	0.0	0.1	0.2	0.2	0.2	0.0	0.0	0.0	0.02	0.1
Feed lime fine	11.9	20.0	20.0	17.6	14.7	12.0	15.0	15.5	15.5	15.5	2.0	6.0	6.0	6.0	6.0
MDCP	8.9	2.0	2.1	2.0	2.0	8.0	2.3	15.7	2.3	2.3	5.8	0.4	0.4	0.5	0.5
Salt fine	2.9	1.8	1.8	1.9	1.9	2.1	1.2	1.2	1.2	1.2	2.6	2.0	2.0	2.0	2.0
Sodium bicarbonate	1.7	2.8	2.6	2.3	1.8	2.1	3.1	3.2	3.15	3.15	2.1	2.7	2.5	2.3	1.9
Choline chloride 60% powder	1.5	0.0	0.0	0.0	0.0	0.0	0.0	0.0	0.0	0.0	0.0	0.0	0.0	0.0	0.0
Avatec 15%	0.2	0.2	0.2	0.2	0.2	0.2	0.2	0.2	0.2	0.2	0.2	0.2	0.2	0.2	0.2
Zinc bacitracin 15%	0.7	0.7	0.7	0.7	0.7	0.7	0.7	0.7	0.7	0.7	0.7	0.7	0.7	0.7	0.7
AxtraPhy broiler (1000FTU)	0.1	0.1	0.1	0.1	0.1	0.1	0.1	0.1	0.1	0.1	0.1	0.1	0.1	0.1	0.1
Broiler premix	3.0	3.0	3.0	3.0	3.0	2.0	2.0	2.0	2.0	2.0	2.0	2.0	2.0	2.0	2.0
Total	1000	1000	1000	1000	1000	1000	1000	1000	1000	1000	1000	1000	1000	1000	1000
Chemical composition															
Dry matter	880.8	895.5	896.4	897.1	898.4	881.3	891.8	894.2	893.7	895.5	882.4	891.2	892.1	893.0	894.8
Crude protein	215.0	215.0	215.0	215.0	215.0	190.0	190.0	190.0	190.0	190.0	181.0	181.0	181.0	181.0	181.0
Ash	42.4	41.5	41.8	42.0	42.6	37.1	35.4	40.8	36.1	36.7	34.2	31.9	32.2	32.4	32.8
Ether extract	42.4	87.6	87.2	87.7	86.9	65.0	85.9	82.0	85.0	84.5	75.5	81.7	81.3	80.8	80.7
Crude fibre	35.9	78.4	77.0	75.1	72.5	30.0	57.1	59.7	56.3	53.3	30.0	55.7	54.3	53.0	49.7
Metabolizable energy (MJ/kg)	11.5	11.5	11.5	11.5	11.5	12.5	12.5	12.5	12.5	12.5	12.8	12.8	12.8	12.8	12.8
Calcium	9.0	10.6	11.2	10.8	10.9	8.4	8.4	11.3	9.6	10.6	9.1	9.5	10.0	10.5	11.5
Total phosphorus	7.7	8.8	8.6	8.5	8.3	7.1	7.8	10.3	7.6	7.3	6.4	7.0	6.8	6.7	6.5
Potassium	9.6	11.1	10.9	10.7	10.4	8.3	9.6	9.1	9.2	8.8	7.9	8.9	8.7	8.5	8.1
Sodium	2.0	2.0	2.0	2.0	2.0	1.8	1.8	1.9	1.8	1.8	2.0	2.0	2.0	2.0	2.0
Chloride	3.0	3.0	3.0	3.0	3.0	2.4	2.4	2.4	2.4	2.4	2.8	2.8	2.8	2.8	2.8

*OMSS* oyster mushroom spent substrate

### Experimental design and animal management

A total of 400 day-old Ross 308 broiler chicks (average initial weight 39.65 ± 0.521 g) purchased from Superior Chicks (Pty) Ltd. (Pretoria, South Africa) were randomly allotted to the 5 dietary treatments (see above), each replicated 8 times with 10 chicks (5 males and 5 females) per pen (size 1.8 m high × 1.5 m long × 1.5 m wide), the experimental unit, for 42 days, in a completely randomised design (CRD). The experiment was carried out in a deep litter system in an environmentally controlled broiler house where the temperature was maintained within the 25 °C and 30 °C range by opening and closing the fitted semi-automatic curtains, depending on daily environmental temperature conditions. The temperature was continuously recorded daily using a thermometer. The curtains were generally opened during the day to allow natural lighting and closed in the evenings until the next morning. However, artificial lighting was provided 24 h a day for the first 2 weeks using electric lights in addition to brooding infra-red lamps (1 per pen). Thereafter, the infra-red lamps were removed, and electric lighting continuously provided for 24 h a day until the end of the experiment. Each pen had 1 feeding trough and 1 drinking trough. On placement in the pens, the chicks were offered StressPack with vitamins and electrolytes for the first 48 h. Otherwise, fresh feed and clean water were offered ad libitum throughout the feeding trial. Daily mortalities were checked in all pens and dead birds removed.

### Performance parameters

Feed offered and leftovers were daily weighed and recorded in all pens in the mornings (08h00 and 10h00) throughout the feeding trial. The daily feed intake was then calculated by subtracting the weight (g) of leftover feed from the weight (g) of feed offered divided by the number of birds per pen. The daily FI values were then converted into weekly averages of FI (g/bird/week) by combining pen averages over 7 days. The broilers were weekly group weighed per pen to get their body weights. Then, weekly BWG (g/bird/week) was calculated by subtracting the previous weekly body weight (g) from the current body weight (g) divided by the number of broilers per pen. Then, FCE was calculated by dividing values of weekly BWG by those of weekly FI.

### Slaughter procedure, carcass characteristics and internal organs

On day 42, 6 birds per pen (3 males and 3 females) were randomly selected and group weighed to get slaughter weight. Then, they were transported to the nearest poultry abattoir at Rooigrond, located ⁓30 km from the experimental site. An hour after arrival, the birds were electrically stunned (70 V) and sacrificed humanely by cervical dislocation whilst unconscious. The jugular vein was cut with a sharp knife at the base of the throat and allowed to bleed for 5 min. Following thorough bleeding, the birds were defeathered and the heads, necks, and feet removed. Visceral organs (liver, spleen, heart, gizzard, and intestine (duodenum, jejunum, ileum, colon, and caecum)) were removed by hand through an opening from the vent to the sternum, weighed individually, and expressed as a percentage of HCW. The carcasses were thoroughly washed and the HCW recorded immediately after slaughter at the abattoir whereas CCW was recorded 24 h after chilling (4 ℃). Carcass cuts (breast, wing, thigh, and drumstick) were then removed by cutting through the joints of the carcass, weighed, and expressed as percentages of the CCW.

### Haemato-biochemistry

A day before slaughter, blood samples were collected in the morning by a trained animal health technician from the wing vain of 2 randomly selected birds per pen into purple-top EDTA-coated vacutainer tubes (for haematology analysis) and red-top Vacuette® Serum Clot Activator tubes without EDTA (for sero-biochemistry analysis). Red blood cells, white blood cells, lymphocytes, heterophils, monocytes, eosinophils, basophils, platelets, haemoglobin, and other haematological parameters were analysed using an automated IDEXX LaserCyte Hematology Analyzer (IDEXX Laboratories, Westbrook, USA) whilst sero-biochemical parameters (glucose, symmetric dimethylarginine (SDMA), phosphate, calcium, total protein, albumin, globulin, albumin/globulin, alanine transaminase, alkaline phosphatase, total bilirubin, cholesterol, amylase, and lipase) were analysed using an automated IDEXX Catalyst One Analyzer (IDEXX Laboratories, Westbrook, USA).

### Meat quality

#### Meat pH and colour

The meat pH, temperature, and colour (L*—lightness, a*—redness, and b*—yellowness) were measured on the breast muscle 45 min and 24 h post-slaughter. A portable waterproof fibre-optic pH and temperature metre (Hanna instruments, HI98163, Romania) was used to measure the pH and temperature. Colour components were determined using a calibrated Konica Minolta colour guide [Narich (Pty) Ltd., Cape Town, RSA]. The colour components were measured during blooming, 30 min after meat samples were removed from the package, which allows oxygenation of myoglobin. The colour measurements were taken on three different locations on the meat surface. The hue angle (H*) and chroma (C*) values were calculated according to the following equations described by the American Meat Science Association [64]:

$${H}^{*}={{\text{tan}}}^{-1}\left(\frac{{b}^{*}}{{a}^{*}}\right)$$

$${C}^{*}=\sqrt{{\left({a}^{*}\right)}^{2}+{\left({b}^{*}\right)}^{2}}$$

#### Water-holding capacity and drip loss

For both water-holding capacity (WHC) and drip loss, 20 g each of meat samples was cut from the breast. The methods described by Honikel (1987) was used for determination of meat WHC using the filter-paper press procedure to determine the capacity of the meat to retain water when subjected to a 60 kg pressure press for 5 min. For drip loss determination, the meat samples were individually hooked inside sample bottles for 72 h and placed in a chiller (4 ℃) (Honikel 1998) whilst ensuring that they do not get in contact with the sides and surfaces of the bottles.

#### Cooking loss and shear force

Twenty-four hours post slaughter, meat samples (40–50 g) were placed inside sealable watertight plastic bags in a water bath and cooked at 85 ℃ for 45 min (Ding et al. 2010). After cooking, they were left to cool-off at room temperature and re-weighed to obtain the new weight post-cooking. Cooking loss (CL) was then calculated by subtracting the weight of meat after cooking from its weight before cooking and then dividing by the weight before cooking and multiplying by 100%. Following cooking and cooling, sub-samples of specified core diameter were cored parallel to the grain of the meat. They were then sheared perpendicular to the direction of the muscle fibres using a Meullenet-Owens’s razor shear blade mounted on a Texture Analyser (Stable Micro System, Model: TA-XT plus, Surrey GU7 1YL, UK). A single shear was performed at the centre of each core with a cross head speed set at 400 mm/min. The mean maximum load (*N*) was computed and recorded for the batch.

### Statistical analysis

All data were analysed using the Statistical Analysis System (SAS 2012) version 9.4. The weekly measured data (weekly FI, BWG, and FCE) were subjected to repeated measures analysis to determine the effects of diet and week and their interaction. In a CRD, all data measured once were statistically analysed for analysis of variance (one-way ANOVA) using the general linear model (GLM) procedure of SAS (SAS 2012) with dietary treatment as an independent variable. Data were presented as Least Square Means (LSMs) with respective pooled SEMs (*n* = 40). The LSMEANS were separated using the probability of difference (PDIFF) option in the LSMEANS statement of SAS and differences among them deemed significant at *P* ≤ 0.05. The statistical model used was *Y*_*ij*_ = *µ* + *α*_*i*_ + *Ɛ*_*ij*_, where *Y*_*ij*_ is the response variable, *α*_*i*_ the effect of diet, and *Ɛ*_*ij*_ the residual error.

## Results

### Performance parameters

The effect of OMSS inclusion in MSC and MSM supplemented diets on overall and weekly FI, BWG, and FCE of broiler chickens is shown in Table 3. Overall, dietary replacement of SBM with combined MSC and MSM decreased FI (*P* < 0.001), BWG (*P* < 0.001), and FCE (*P* < 0.001) regardless of the week post-hatching of birds and the deleterious effects were not abrogated by dietary inclusion of OMSS (*P* > 0.05). Notwithstanding, the repeated measures results revealed a significant diet × week interaction effect on FI (*P* < 0.001), BWG (*P* < 0.001), and FCE (*P* < 0.001), with dietary inclusion of 1.25% OMSS eliciting limited improvements in FI in weeks 4 and 6, BWG in weeks 3, 5 and 6, and FCE in weeks 3 and 5.

**Table 3:** Effects of oyster mushroom spent substrate inclusion in marula seed cake and mucuna seed meal supplemented diets on weekly and overall feed intake, body weight gain, and feed conversion efficiency of broiler chickens

Parameter	Week				Treatment		SEM		*P* value	
		SBM	MSC + MSM							
			Only	+ 1.25%OMSS	+ 2.5%OMSS	+ 5%OMSS		Diet	Week	Diet × week
FI (g/bird)	1	127.75^a^	101.58^b^	98.94^b^	93.50^b^	94.80^b^	2.999	*P* < 0.001		
	2	306.75^a^	216.44^b^	219.71^b^	208.66^b^	240.65^b^	18.712	*P* < 0.01		
	3	473.44^a^	324.77^b^	345.04^b^	281.73^c^	318.96^b^	14.238	*P* < 0.001	*P* < 0.001	*P* < 0.001
	4	709.63^a^	485.47^c^	547.00^b^	390.80^d^	489.34^bc^	20.127	*P* < 0.001		
	5	993.50^a^	748.76^bc^	803.25^b^	565.83^d^	712.03^c^	27.410	*P* < 0.001		
	6	1144.19^a^	904.57^c^	1033.07^b^	757.34^d^	896.81^c^	25.847	*P* < 0.001		
BWG (g/bird)	1	92.31^a^	53.84^b^	52.56^b^	47.53^b^	48.88^b^	2.34	*P* < 0.001		
	2	242.56^a^	111.57^b^	124.38^b^	112.96^b^	116.49^b^	8.026	*P* < 0.001		
	3	283.19^a^	121.79^c^	156.36^b^	101.04^c^	138.40^bc^	9.612	*P* < 0.001	*P* < 0.001	*P* < 0.001
	4	452.25^a^	229.76^b^	271.20^b^	152.09^c^	244.57^b^	15.110	*P* < 0.001		
	5	611.75^a^	365.81^c^	431.00^b^	294.57^d^	390.54^bc^	19.504	*P* < 0.001		
	6	877.06^a^	494.49^c^	584.44^b^	460.51^c^	536.62^bc^	25.259	*P* < 0.001		
FCE	1	0.72^a^	0.53^b^	0.53^b^	0.51^b^	0.52^b^	0.016	*P* < 0.001		
	2	0.79^a^	0.52^b^	0.57^b^	0.54^b^	0.53^b^	0.027	*P* < 0.001		
	3	0.59^a^	0.38^c^	0.45^b^	0.36^c^	0.43^bc^	0.014	*P* < 0.001	*P* < 0.001	*P* < 0.001
	4	0.64^a^	0.47^b^	0.49^b^	0.39^c^	0.50^b^	0.014	*P* < 0.001		
	5	0.61^a^	0.49^c^	0.54^b^	0.52^b^	0.55^b^	0.014	*P* < 0.001		
	6	0.77^a^	0.55^b^	0.57^b^	0.61^b^	0.60^b^	0.024	*P* < 0.001		
Overall FI (g/bird)		3755.25^a^	2781.59^c^	3046.99^b^	2297.86^d^	2752.64^c^	92.058	*P* < 0.001		
Overall BWG (g/bird)		2559.13^a^	1377.25^c^	1619.94^b^	1168.69^d^	1475.50^bc^	63.583	*P* < 0.001		
Overall FCE		0.68^a^	0.49^c^	0.53^bc^	0.51^c^	0.54^b^	0.012	*P* < 0.001		

Means in the same row with different superscripts (^abcd^) are significantly different

*BWG* body weight gain, *FCE* feed conversion efficiency, *FI* feed intake, *MSC* marula seed cake, *MSM* mucuna seed meal, *OMSS* oyster mushroom spent substrate, *SBM* soya bean meal, *SEM* standard error of the mean

### Carcass characteristics and internal organs

The effects of OMSS inclusion in MSC and MSM supplemented diets on carcass characteristics and internal organs of broiler chickens are presented in Table 4 and 5, respectively. Data showed that dietary inclusion of MSC and MSM decreased the weight of birds at slaughter (*P* < 0.001), as well as their HCW (*P* < 0.001), CCW (*P* < 0.001), breast weights (*P* < 0.001), and back lengths (*P* < 0.001). Interestingly, dietary incorporation of 1.25% OMSS reversed these deleterious effects of MSC and MSM on slaughter weight (*P* < 0.001), HCW (*P* < 0.001), and CCW (*P* < 0.001), though to a limited extent, whilst higher levels of the spent substrate worsened them. Hence, birds fed SBM-supplemented diets had the heaviest slaughter weights, HCW, CCW, breast weights and back lengths compared to those on diets supplemented with MSC and MSM with and without OMSS (*P* < 0.001). Intriguingly, dietary MSC and MSM increased the thigh (*P* < 0.001) and wing (*P* < 0.01) weights, particularly when combined with 2.5% OMSS.

**Table 4:** Effects of oyster mushroom spent substrate inclusion in marula seed cake and mucuna seed meal-supplemented diets on carcass characteristics (% of CCW, unless stated otherwise) of broiler chickens

Parameter	Treatment					SEM	*P* value
	SBM	MSC + MSM					
		Only	+ 1.25%OMSS	+ 2.5%OMSS	+ 5%OMSS		
SW (g/bird)	2599.06^a^	1416.88^c^	1659.69^b^	1208.44^d^	1514.69^bc^	63.751	*P* < 0.001
HCW (g)	1920.00^a^	942.50^c^	1153.75^b^	805.00^c^	1066.25^bc^	72.81	*P* < 0.001
CCW (g)	1880.00^a^	935.00^c^	1148.75^b^	792.50^c^	1052.50^bc^	73.761	*P* < 0.001
Dressing %	73.42	66.44	69.47	66.40	70.49	2.616	0.2972
Breast wt (%)	24.37^a^	15.97^b^	18.14^b^	15.14^b^	17.24^b^	1.054	*P* < 0.001
Drumstick wt (%)	6.62	7.44	7.10	7.25	7.30	0.277	0.2780
Thigh wt (%)	7.75^c^	9.59^ab^	9.11^ab^	9.91^a^	8.96^b^	0.322	*P* < 0.001
Wing wt (%)	5.27^b^	6.23^ab^	5.92^ab^	6.45^a^	5.81^b^	0.209	*P* < 0.01
Back length (cm)	16.88^a^	14.84^bc^	15.44^b^	13.99^c^	14.35^bc^	0.451	*P* < 0.001

Means in the same row with different superscripts (^abc^) are significantly different

*CCW* cold carcass weight, *HCW* hot carcass weight, *MSC* marula seed cake, *MSM* mucuna seed meal, *OMSS* oyster mushroom spent substrate, *SBM* soya bean meal, *SEM* standard error of the mean, *wt* weight, *SW* slaughter weight

**Table 5:** Effects of oyster mushroom spent substrate inclusion in marula seed cake and mucuna seed meal-supplemented diets on weights and lengths of internal organs (% of HCW, unless stated otherwise) of broiler chickens

Parameter	Treatment					SEM	*P* value
	SBM	MSC + MSM					
		Only	+ 1.25%OMSS	+ 2.5%OMSS	+ 5%OMSS		
Liver wt (%)	2.39^c^	3.17^b^	3.03^b^	3.79^a^	3.17^b^	0.186	*P* < 0.001
Spleen wt (%)	0.17	0.21	0.19	0.24	0.16	0.019	0.0562
Proventriculus wt (%)	0.61^b^	1.29^a^	1.14^a^	1.24^a^	1.16^a^	0.080	*P* < 0.001
Gizzard wt (%)	2.39^c^	4.06^a^	3.13^b^	3.78^ab^	3.75^ab^	0.244	*P* < 0.001
Duodenum wt (%)	1.02^b^	2.02^a^	1.84^a^	2.12^a^	1.74^a^	0.138	*P* < 0.001
Jejunum wt (%)	1.74^b^	2.99^a^	2.80^a^	2.99^a^	2.92^a^	0.163	*P* < 0.001
Ileum wt (%)	1.38^b^	2.33^a^	2.15^a^	2.44^a^	2.19^a^	0.116	*P* < 0.001
Caecum wt (%)	0.89^b^	1.08^b^	1.30^ab^	1.50^a^	1.15^b^	0.108	*P* < 0.01
Colon wt (%)	0.16	0.27	0.31	0.17	0.18	0.049	0.1207
Duodenum length (cm)	33.04	32.46	34.43	30.97	33.25	1.064	0.2521
Jejunum length (cm)	80.71	80.58	80.84	75.94	78.42	2.166	0.4397
Ileum length (cm)	80.70	78.51	80.14	76.59	76.27	2.622	0.6749
Caecum length (cm)	21.14	19.04	19.68	17.93	18.71	0.593	0.0760
Colon length (cm)	5.64	5.71	5.17	4.36	4.78	0.493	0.2676

Means in the same row with different superscripts (^abc^) are significantly different

*MSC* marula seed cake, *MSM* mucuna seed meal, *OMSS* oyster mushroom spent substrate, *SBM* soya bean meal, *SEM* standard error of the mean, *wt* weight

In terms of internal organs, whilst there was no effect of diet on spleen and colon weights as well as lengths of the small and large intestines and caecum (*P* > 0.05), dietary inclusion of MSC and MSM increased the weights of the liver (*P* < 0.001), proventriculus (*P* < 0.001), gizzard (*P* < 0.001), duodenum (*P* < 0.001), jejunum (*P* < 0.001), ileum (*P* < 0.001), and caecum (*P* < 0.01). These increases were not reversed by dietary inclusion of OMSS in all internal organs (*P* > 0.05), except in the case of gizzard weight where inclusion of 1.25% OMSS had an ameliorative effect (*P* < 0.001). Otherwise, higher inclusion levels of OMSS exacerbated the increase in the weights of internal organs as reflected particularly in the liver (*P* < 0.001), gizzard (*P* < 0.001) and caecum (*P* < 0.01).

### Haemato-biochemistry

Tables 6 and 7, respectively, show effects of OMSS incorporation in MSC and MSM-supplemented diets on haematology and serum biochemistry of broiler chickens. There was no effect of dietary treatment on all haematological parameters of the birds (*P* > 0.05). However, dietary MSC and MSM decreased serum SDMA (*P* < 0.05) and alanine transaminase activity (*P* < 0.05) whilst it increased serum alkaline phosphatase (*P* < 0.05). Similarly, OMSS inclusion further quadratically decreased serum SDMA and alanine transaminase, with the lowest responses at 2.5% OMSS inclusion level, whilst it abrogated the increase in serum alkaline phosphatase, particularly at 5% OMSS inclusion level (*P* < 0.05). Otherwise, dietary replacement of SBM with MSC and MSM quadratically increased broiler chicken serum cholesterol concentrations whilst OMSS further augmented this increase with maximum responses at 2.5% OMSS inclusion level (*P* < 0.01).

**Table 6:** Effects of oyster mushroom spent substrate inclusion in marula seed cake and mucuna seed meal-supplemented diets on haematological parameters of broiler chickens

Parameter	Treatment					SEM	*P* value
	SBM	MSC + MSM					
		Only	+ 1.25%OMSS	+ 2.5%OMSS	+ 5%OMSS		
Red blood cells (× 10^12^/L)	0.42	0.42	0.96	0.42	0.43	0.233	0.3855
Haemoglobin (g/dL)	14.52	9.69	10.01	9.84	9.40	2.154	0.4220
White blood cells (× 10^9^/L)	289.60	300.00	282.14	299.06	298.66	8.135	0.4640
Heterophils (× 10^9^/L)	23.75	27.61	45.86	24.42	23.54	8.602	0.3152
Lymphocytes (× 10^9^/L)	216.36	234.03	229.34	249.22	249.68	17.27	0.6195
Monocytes (× 10^9^/L)	127.84	132.88	135.08	142.95	142.40	8.35	0.6680
Eosinophils (× 10^9^/L)	2.42	2.17	2.58	2.62	2.85	0.225	0.3003
Basophils (× 10^9^/L)	0.10	0.10	0.21	0.1	0.1	0.053	0.4651
Platelet (K/µL)	2500.00	2371.25	2180.37	2500.00	2500.00	134.553	0.3792

*MSC* marula seed cake, *MSM* mucuna seed meal, *OMSS* oyster mushroom spent substrate, *SBM* soya bean meal, *SEM* standard error of the mean

**Table 7:** Effects of oyster mushroom spent substrate inclusion in marula seed cake and mucuna seed meal-supplemented diets on serum biochemistry of broiler chickens

Parameter	Treatment					SEM	*P* value
	SBM	MSC + MSM					
		Only	+ 1.25%OMSS	+ 2.5%OMSS	+ 5%OMSS		
Glucose (mg/dL)	153.06	161.81	160.25	166.69	165.19	6.63	0.6350
SDMA (µg/dL)	20.56^a^	17.75^ab^	16.31^b^	16.06^b^	19.19^a^	1.177	*P* < 0.05
Phosphate (mg/dL)	7.81	6.73	6.71	6.72	6.99	0.299	0.0588
Calcium (mg/dL)	9.10	9.23	9.06	9.28	9.41	0.174	0.6150
Total protein (g/dL)	3.52	3.11	3.08	3.29	3.31	0.180	0.4448
Albumin (g/dL)	1.32	1.24	1.17	1.21	1.28	0.050	0.2629
Globulin (g/dL)	2.21	1.88	1.91	2.09	2.08	0.143	0.4626
Albumin/globulin	0.60	0.58	0.62	0.58	0.61	0.015	0.2764
Alanine transaminase (U/L)	24.44^a^	15.63^b^	13.00^b^	11.88^b^	14.25^b^	2.646	*P* < 0.05
Alkaline phosphatase (U/L)	346.63^b^	747.63^a^	558.50^ab^	590.77^ab^	490.25^b^	83.965	*P* < 0.05
Total bilirubin (mg/dL)	0.57	0.26	0.20	0.18	0.20	0.156	0.3731
Cholesterol (mg/dL)	93.06^b^	107.75^a^	111.63^a^	119.94^a^	110.82^a^	4.600	*P* < 0.01
Amylase (U/L)	333.81	231.63	279.50	185.88	236.56	39.349	0.1141
Lipase (U/L)	251.56	284.69	236.63	192.88	230.75	27.660	0.2375

Means in the same row with different superscripts (^abc^) are significantly different

*MSC* marula seed cake, *MSM* mucuna seed meal, *OMSS* oyster mushroom spent substrate, *SBM* soya bean meal, *SDMA* symmetric dimethylarginine, *SEM* standard error of the mean

### Meat quality

The influence of including OMSS in MSC and MSM-supplemented diets on meat quality of broiler chickens is shown in Table 8. At 45 min post-slaughter, there were no effects of diet on the pH, temperature, lightness, yellowness, and chroma of meat (*P* > 0.05). However, dietary replacement of SBM with MSC and MSM increased the redness of meat and this effect was reversed by dietary inclusion of OMSS particularly at 1.25% inclusion level (*P* < 0.01). In contrast, at the same time post-slaughter, SBM replacement with MSC and MSM decreased meat hue angle whilst OMSS reversed the effect particularly when included at 2.5% level (*P* < 0.05).

**Table 8:** Effects of oyster mushroom spent substrate inclusion in marula seed cake and mucuna seed meal-supplemented diets on meat quality of broiler chickens

Parameter	Treatment					SEM	*P* value
	SBM	MSC + MSM					
		Only	+ 1.25%OMSS	+ 2.5%OMSS	+ 5%OMSS		
pH_45 min_	5.91	5.85	5.85	5.82	8.81	0.028	0.1374
Temperature_45 min_ (°C)	29.65	27.61	27.32	26.82	28.39	0.570	0.1230
L*_45 min_	53.85	54.00	52.63	54.41	52.45	0.830	0.3685
a*_45 min_	2.31^b^	3.47^a^	2.66^b^	3.36^ab^	3.43^ab^	0.271	*P* < 0.01
b*_45 min_	3.96	2.85	2.79	3.74	3.09	0.413	0.1837
H*_45 min_ (°)	0.99^a^	0.69^b^	0.78^b^	0.83^ab^	0.73^b^	0.071	*P* < 0.05
C*_45 min_	21.11	11.98	11.32	18.39	13.42	2.908	0.0913
pH_24 h_	5.89^a^	5.82^b^	5.83^b^	5.83^b^	5.80^b^	0.014	*P* < 0.01
Temperature_24 h_ (°C)	13.96	13.16	13.90	12.47	14.70	0.775	0.3257
L*_24 h_	52.20	52.66	51.77	53.37	51.73	0.658	0.3821
a*_24 h_	3.84	4.08	3.62	3.79	3.98	0.244	0.7284
b*_24 h_	5.82	5.11	4.73	5.50	4.74	0.327	0.0952
H*_24 h_ (°)	0.99	0.90	0.91	0.96	0.87	0.038	0.1987
C*_24 h_	38.83	31.13	26.06	34.92	27.07	3.639	0.0934
Cooking loss (%)	36.71	40.98	32.38	43.29	36.68	3.426	0.2148
Drip loss (%)	9.88	8.06	8.70	10.49	9.05	0.890	0.3417
WHC (%)	86.07	84.30	83.53	82.83	84.53	1.556	0.6573
Shear force (N)	6.02^ab^	6.75^ab^	5.41^b^	7.33^a^	6.65^ab^	0.467	*P* < 0.05

Means in the same row with different superscripts (^abc^) are significantly different

L* lightness, a* redness, b* yellowness, C* chroma, H* hue angle, *MSC* marula seed cake, *MSM* mucuna seed meal, *OMSS* oyster mushroom spent substrate, *SBM* soya bean meal, *SEM* standard error of the mean, *WHC* water-holding capacity

At 24 h post-slaughter, whilst dietary replacement of SBM with MSC and MSM did not influence most meat quality components (*P* > 0.05), it decreased meat pH (*P* < 0.01), with OMSS inclusion having no influence on this response. On the other hand, at the same time post-slaughter, dietary substitution of SBM with MSC and MSM increased meat shear force, with this effect however ameliorated by 1.25% OMSS whilst worsened by higher inclusion levels of the spent substrate (*P* < 0.05).

## Discussion

The observed decreased performance and carcass traits in response to dietary combined MSC and MSM mimic previous findings from studies with broiler chickens fed diets supplemented with ANF-bound unconventional protein sources (Miya et al. 2019; Qaisrani et al. 2020; Sugiharto et al. 2021; Liebl et al. 2022; Mthana et al. 2023). This is not unexpected as MSC can be infested with low levels of mycotoxins DON and T-2 toxin as well as lipid peroxides (Mthiyane and Mhlanga 2017) whilst MSM is fraught with numerous antinutritional compounds including a high content of L-DOPA (Pugalenthi and Vadivel 2007; Barman et al. 2018). Consumption of diets containing DON and T-2 toxin (Huff et al. 1986; Kubena et al. 1988, 1989), oxidised lipids (Engberg et al. 1996) and toxic phytochemicals including L-DOPA (Del Carmen et al. 1999; Vadivel and Pugalenthi 2010) has previously induced detrimental effects on broiler chicken FI, BWG, FCE and carcass characteristics through various mechanisms including oxidative stress (Engberg et al. 1996; Weber et al. 2010; Nikolova et al. 2019).

Meanwhile, the intriguingly increased broiler chicken thigh and wing weights in response to dietary MSC and MSM are unprecedented but speculated to be associated with the presence of oleic acid in MSC and L-DOPA in MSM. Indeed, oleic acid induces proliferative effects in muscle cells (Rao et al. 1995; Lu et al. 1998; Belal et al. 2018) whilst L-DOPA is a precursor for dopamine, epinephrine and norepinephrine, the catecholamines that possess muscle accretion attributes (Navegantes et al. 2000). Hence, dietary consumption of MSM induces anabolic effects and increases muscle mass through L-DOPA increasing concentrations of growth hormone (Alleman et al. 2011), testosterone (Suresh and Prakash 2012) and dopamine (Lieu et al. 2012). For this reason, *M. pruriens* seeds have been used as a dietary supplement for body-building in humans (Alleman et al. 2011) and meat production in beef cattle (Yantika et al. 2016).

Also, the increased weights of the liver and digestive organs in response to dietary MSC and MSM corroborate previous studies with broiler chickens fed diets supplemented with combined (Liebl et al. 2022) and single APS including MSM (Mthana and Mthiyane 2023) and leguminous leaf meals (Miya et al. 2019). The above-mentioned mechanism of ANFs appears to also underlie the increased weights of the essential metabolic and digestive organs. In particular, the increased liver weight suggested inflammation of this metabolic organ as it attempted to biodegrade toxic dietary chemicals (Poudyal et al. 2010; Adamu et al. 2012). This is congruent with the observation of increased serum alkaline phosphatase activity in response to dietary MSC and MSM, which indicated liver damage in line with previous studies involving APS (Adamu et al. 2012). Notwithstanding, dietary fibre, which was about 2 × more in MSC and MSM-supplemented diets, in comparison to those supplemented with SBM (Table 2), would also have contributed to the increased weights of digestive organs consequent to stimulation of goblet cell proliferation (Murai et al. 2018) as an adaptive mechanism to enhance nutrient digestion and absorption (Rezaei et al. 2018; Jha and Mishra 2021).

On the other hand, the decreased serum alanine transaminase and SDMA levels in contrast to increased serum alkaline phosphatase following dietary consumption of MSC and MSM suggest that these APS induced not so much liver and kidney injury as they affected bone resorption. Whilst alanine transaminase is primarily a biomarker for liver function (Washington and van Hoosier 2012), alkaline phosphatase is a key indicator of bone metabolism (LeBoff et al. 2022) with its serum levels negatively correlated with bone mineral density (Cheng and Zhao 2023). Hence, the observed increased serum alkaline phosphatase in contrast to decreased serum alanine transaminase levels may indicate that dietary consumption of MSC and MSM induced bone loss in the birds. Indeed, treatment with L-DOPA, the main bioactive compound in MSM, has been associated with osteoporosis in mice (Ung et al. 2012) probably through L-DOPA breakdown by catechol-O-methyltransferase inducing formation of homocysteine (Postuma and Lang 2004), a high systemic level of which causes osteoporosis and bone fractures mediated by impaired collagen cross-linking (Van Meurs et al. 2004). Otherwise, considering its biomarker status in acute kidney injury (Kielstein et al. 2006), oxidative stress and inflammation (Tain and Hsu 2017), the observed decreased SDMA levels in response to dietary MSC and MSM portrayed these APS to have caused no damage to the kidney nor any physiological stress to the broiler chickens.

Otherwise, the increased serum cholesterol concentrations in response to dietary MSC and MSM consumption confirm our previous findings with broiler chickens fed MSC-supplemented diets (Mthana et al. 2023). As argued by Mthana et al. (2023), the increased serum cholesterol is likely induced by the high residual oil content of oleic acid in MSC. In this regard, a previous study demonstrated dietary intake of oleic acid-rich olive oil to improve blood plasma and adipose tissue levels of health-beneficial high-density lipoprotein cholesterol (HDL-C) (Nogoy et al. 2020). Oleic acid selectively increases systemic levels of HDL-C as it decreases those of its chronic heart disease-associated low-density lipoprotein cholesterol (LDL-C) counterpart (Rudel et al. 1995; Kwon and Choi 2015; Nogoy et al. 2020). Unfortunately, there are currently no comparable literature data on serum cholesterol responses to dietary MSC and MSM supplementation in broiler chickens.

In terms of meat quality, the increased redness in contrast to decreased hue angle of broiler meat at 45 min post-slaughter may be a response to dietary MSC. Corroborating our results, Mazizi et al. (2020) also observed increased breast meat redness in Japanese quails fed incremental dietary levels of MSC at 24 h post-slaughter whilst Mthana et al. (2022) observed no colour changes in meat from broiler chickens fed MSM-supplemented diets. The increased redness may have been induced by the high iron and protein contents of MSC (Malebana et al. 2018) that would have enhanced the meat myoglobin content. Constituting the most protein in meat, myoglobin contains heme–iron which gives meat its reddish colour (Buzala et al. 2016). Indeed, Lin et al. (2020) observed increased breast meat redness in broiler chickens fed diets with incremental dietary levels of iron. On the other hand, the decreased meat hue angle at 45 min post-slaughter conflicts with the observations of Mazizi et al. (2020) who demonstrated increased breast meat hue angle at 30 min post-slaughter in Japanese quails fed incremental dietary levels of MSC. Indicating meat brightening into a reddish colour, it is speculated that the decreased meat hue angle was a response to the presence of oleic acid in MSC. Oleic acid is a potent antioxidant (Bhattacharjee et al. 2020) that would have reduced the meat metmyoglobin to myoglobin and oxymyoglobin, leading to bright reddish colour of the birds’ meat.

The decrease in meat pH in contrast to the increase in its shear force at 24 h post-slaughter in response to dietary MSC and MSM was probably due to high glycogen reserves in broiler muscles at slaughter. The high glycogen levels, likely due to the high residual oil content of MSC, would have increased the lactic acid production post rigor mortis and thus decreased meat pH (Uhlířová et al. 2018). Indeed, studies have previously demonstrated increased muscle glycogen levels following dietary fat intake (Harking et al. 1992; Jones et al. 1992; Scott et al. 1992). In agreement with the observed increased meat redness at 45 min post-slaughter in response to dietary MSC and MSM, our data corroborate previous reports that meat colour gets brighter as its pH decreases (Strzyżewski et al. 2008; Bocian et al. 2015). Otherwise, the increased meat shear force—an indicator of decreased meat tenderness—as its pH decreased, concurs with previous reports of decreased meat tenderness in the early post-mortem period at pH 5.8–6.3 (Yu and Lee 1986).

In terms of OMSS, the general lack of abrogative or limited ameliorative effects of the spent substrate on the deleterious effects of MSC and MSM on performance parameters, slaughter weight, carcass characteristics, and internal organs can be ascribed to the OMSS-induced high dietary fibre content. In this regard, due to their richness in fibre with almost 30% cellulose and about 26% lignin (De’Nobili et al. 2021), sunflower husks were used as a growing medium for the white-rot fungi in this study. Hence, the residual fibre remaining as part of the OMSS was apparently too high and would seem to have served as an ANF that adversely affected FI, nutrient digestibility and absorption as well as growth performance as has been previously reported (Meremikwu et al. 2013; Jha and Mishra 2021). For this reason, dietary inclusion of OMSS at levels higher than 1.25% exacerbated the increase in the weights of internal organs particularly the liver, gizzard, and caecum in this study.

Nonetheless, showing a significant diet × week interaction effect on FI, BWG, and FCE, with dietary inclusion of 1.25% OMSS eliciting some improvements in FI in weeks 4 and 6, BWG in weeks 3, 5 and 6, and FCE in weeks 3 and 5, our data indicated that the spent substrate was more effective in ameliorating the deleterious effects of MSC and MSM in older rather than younger (< 3 weeks old) broiler chicks. Indeed, the digestive system of young broiler chicks during the first 2 weeks post-hatching (i.e. starter phase) is immature and physiologically inefficient in digesting and absorbing nutrients (Jha and Mishra 2021). However, upon chick attainment of physiological maturity post the starter phase, dietary inclusion of a low level of 1.25% OMSS evidently enhanced performance and carcass traits whilst it ameliorated the increase in gizzard weight consequent to the improved bird ability to secrete digestive enzymes and acquisition of a diverse and functional microbiota to ferment fibre-rich feed (Ravindran and Abdollahi 2021). Also, the extracellular oxidoreductase enzymes, assumed to have been abundantly secreted by the oyster mushroom mycelium onto the OMSS, have potent antioxidant properties (Elkhateeb et al. 2022) with ability to bolster the antioxidant capacity of birds fed the spent substrate (Chuang et al. 2020) and would therefore appear to have effectively quenched the oxidative stress induced by the noxious chemical elements in MSC and MSM. Our data corroborate previous findings by Lee et al. (2012) and Chuang et al. (2020) who observed improved FCE, intestinal barrier function, nutrient uptake, and toll-like receptor 4 (TLR4) levels in broiler chickens fed diets incorporated with low (0.5% and 1%) rather than high (> 1%) levels of OMSS.

The observed lack of effect of dietary OMSS on all haematological parameters suggests the safety of the spent substrate as a broiler chicken feed additive and corroborates previous studies with the modern birds and geese (Ullah et al. 2020; Klausen et al. 2023). Indeed, the OMSS is a sterile by-product from the cultivation of oyster mushrooms, a safe human food that is not only rich in nutrients but also a plethora of low-molecular weight bioactive compounds and antioxidant enzymes (Corrêa et al. 2016; Rizzo et al. 2021) that are secreted and deposited onto the spent substrate (Klausen et al. 2023). Further demonstrating its safety for broiler chickens, dietary OMSS intake augmented the MSC and MSM-induced decrease in serum SDMA and alanine transaminase as it abrogated the increase in serum alkaline phosphatase. Since SDMA is a biomarker for acute kidney injury (Kielstein et al. 2006), oxidative stress and inflammation (Tain and Hsu 2017) whilst alanine transaminase indicates liver function (Hassan et al. 2020) and alkaline phosphatase bone resorption (LeBoff et al. 2022; Cheng and Zhao 2023), our data suggest that the OMSS further ameliorated possible damage of the essential metabolic organs, bone resorption, and physiological stress that could have been induced by MSC and MSM-bound ANFs. Meanwhile, whilst dietary OMSS was envisaged to decrease serum cholesterol levels as has been previously observed (Hassan et al. 2020), no explanation could be found for the positive effect of the fungal spent substrate on this blood parameter.

The mechanism underlying the OMSS reversal of MSC and MSM induced increase in redness and decrease in hue angle of meat is not known. It is however possible that this is due to the relatively lower OMSS content of iron (Table 1), the mineral that is required for improving muscle myoglobin content and hence meat redness (Lin et al. 2020). In a previous study, Chang et al. (2016) observed a higher colour score in meat from geese fed diets supplemented with OMSS, though these researchers did not specifically measure meat redness. Regarding the meat hue angle, it is possible that the inclusion of the OMSS diluted the dietary content of oleic acid, the antioxidant MUFA (Bhattacharjee et al. 2020) with potential to reduce meat metmyoglobin to myoglobin and oxymyoglobin, resulting in improved meat redness.

Otherwise, regarding meat shear force, it is evident that the OMSS contained bioactive compounds that induced meat tenderness particularly when it was included in the diet at 1.25% level. Indeed, previous corroborative studies demonstrated that addition of *Cordyceps militaris* mushroom onto samgyetang meat not only improved its flavour but also its tenderness (Barido et al. 2020) whilst *C. militaris* mushroom-derived exogenous protease introduction into spent hen breast muscle improved its texture (Barido and Lee 2021). However, it appears that these OMSS-bound bioactive compounds and enzymes were diluted, probably by fibre, at higher dietary inclusion levels of the spent substrate, resulting in higher shear force values.

In conclusion, our data showed that dietary replacement of SBM with combined MSC plus MSM induced detrimental effects on broiler chicken performance, carcass traits, internal organs, haemato-biochemistry, and meat quality, which were however limitedly abrogated by 1.25% inclusion level of the OMSS. There is a need for investigation of mushroom-mediated solid-state fermentation of combined MSC and MSM and their inclusion in broiler diets in future studies.

## Data Availability

The data used in supporting the conclusions of this article will be made available by the corresponding author, without undue reservation.

## Declarations

### Disclaimer

The funders had no role in the design of the study; in the collection, analyses, or interpretation of data; in the writing of the manuscript; or in the decision to publish the results.
